# “Against Shameless and Systematic Calumny”: Strategies of Domination and Resistance and Their Impact on the Bodies of the Poor in Nineteenth-Century Ireland

**DOI:** 10.1007/s41636-019-00219-2

**Published:** 2020-01-14

**Authors:** Jonny Geber, Barra O’Donnabhain

**Affiliations:** 1 School of History, Classics and Archaeology, University of Edinburgh, William Robertson Wing, Old Medical School, Teviot Place, Edinburgh, EH8 9AG UK; 2 Department of Archaeology, University College Cork, Cork, T12 CY82 Ireland

**Keywords:** bioarchaeology, colonialism, identity, prison, workhouse

## Abstract

Mid-Victorian British characterizations of Ireland and much of its population blamed race and “moral character” for the widespread poverty on the island. The Irish poor were portrayed as a “race apart” whose inherent failings were at least partly to blame for the mortality they suffered during the Great Famine of 1845–1852. Recent excavations at Kilkenny workhouse and Spike Island convict prison have produced skeletal assemblages from this critical period. These collections have enabled bioarchaeological analysis of parameters mentioned by the Victorians as indicative of the distinctiveness of the Irish poor: stature, interpersonal violence, and tobacco use. Bioarchaeological data indicate that the differences between Irish and British populations in stature and risk of violence were exaggerated. Such characterizations, we argue, were part of a strategy of “Othering” that served to legitimize colonial domination. This exertion of power did not go uncontested, as the pattern of tobacco use may be indicative of forms of passive resistance.

## Extracto

Las caracterizaciones británicas a mediados de la época victoriana de Irlanda y gran parte de su población echaban la culpa a su raza y “carácter moral” por la pobreza generalizada en la isla. Los pobres irlandeses fueron retratados como una “raza aparte”, cuyas fallas inherentes fueron al menos en parte culpables de la mortalidad que sufrieron durante la Gran Hambruna de 1845–1852. Las excavaciones recientes en la casa de trabajo de Kilkenny y la prisión de convictos de Spike Island han producido ensamblajes esqueléticos de este período crítico. Estas colecciones han permitido el análisis bioarqueológico de los parámetros mencionados por los victorianos como indicativos del carácter distintivo de los pobres irlandeses: estatura, violencia interpersonal y consumo de tabaco. Los datos bioarqueológicos indican que las diferencias entre las poblaciones irlandesas y británicas en áreas de estatura y riesgo de violencia fueron exageradas. Tales caracterizaciones, argumentamos, fueron parte de una estrategia de “Otredad” que sirvió para legitimar la dominación colonial. Este ejercicio de poder no se realizaba sin oposición, ya que el patrón de consumo de tabaco puede ser indicativo de formas de resistencia pasiva.

## Résumé

Les descriptions de l’Irlande et de la plupart de sa population par la société Britannique du milieu de l’ère victorienne, blâmaient la race et le « caractère moral » pour expliquer la pauvreté généralisée sur l’île. Les Irlandais pauvres étaient décrits comme une « race à part », dont les faiblesses inhérentes étaient à tout le moins et en partie la raison de la mortalité les ayant frappés durant la Grande famine de 1845–1852. Les récentes fouilles archéologiques à l’hospice des pauvres de Kilkenny et dans la prison de Spike Island ont mis au jour des assemblages ostéologiques de cette période critique. Ces collections ont permis une analyse bioarchéologique des paramètres mentionnés par les Victoriens comme indicatifs du caractère distinctif de l’Irlandais pauvre: stature, violence interpersonnelle et consommation de tabac. Les données bioarchéologiques indiquent que les différences entre les populations irlandaise et britannique étaient exagérées quant aux traits liés à la stature et au risque de violence. Ces caractérisations faisaient partie selon nous d’une stratégie d’« Altérisation » servant à légitimer la domination coloniale. L’exercice de ce pouvoir n’a pas manqué d’être contesté, comme l’indiquent les usages de consommation du tabac qui ont pu représenter une forme de résistance passive.

When the great lord passes the wise peasant bows deeply and silently farts.

—Ethiopian proverb (Scott 1990)

## Introduction

Whether or not Ireland can be viewed as a colony has been the matter of debate among historians (McDonough 2005; Howe 2008). The question defies a simple answer, in part, because of the fact that colonialism has taken many different forms over time and provoked diverse reactions around the world. The form of colonialism that developed with the rise of capitalism resulted from the ideology of imperialism, which has been defined as “the extension and expansion of trade and commerce under the protection of political, legal and military controls” (Childs and Williams 1997:227). In the attempt to control the indigenous inhabitants of an occupied area, unequal relations of power were usually constructed between colonizer and colonized. This inequality was often legitimized by narratives of racial distinction that emphasized the supposed inherent failings of the colonized and a “natural order” that was hierarchical. This, in turn, resulted in an elision of race and poverty that was understood as justifying colonial enterprises rather than being their product. Orser (2011) has argued that this connection between race and poverty has been a feature of European colonialism in the modern era. While most definitions of colonial enterprises place an emphasis on economic and political relationships, usually in the context of the settlement of a group of people in a new location, Orser also noted how the operation of colonialism can be considered at multiple levels. In the 1950s, for example, Fanon (1952) wrote about the psychological impact of colonialism on subjugated populations, while, more recently, Stoler (2002) has considered the role of the intimate in the negotiation and maintenance of colonial relationships. These perspectives place bodies, both settler and indigenous, at the center of colonial discourse. This study uses bodies, in the form of archaeological skeletal remains, to explore expressions of lower-class identity in 19th-century Ireland in light of common perceptions of the “Irish race” and “national character” held by middle- and upper-class Victorian society in Britain that played an important role in the relationship between the two islands.

Modern Ireland has been shaped by its relationship with Britain. The experience of imperial control has been a dominant factor in the production of culture in the former, including contemporary identities and narratives of the past. In political, social, and demographic terms, modern Ireland has been shaped by the drastic changes that took place in the 19th century. Perhaps the greatest of these was the loss of nearly 50% of the population of the island in the 70 years between 1841 and 1911. Today, Ireland is the only country in Europe in which the modern population is significantly lower than it was in the early 1800s. The 19th century had begun with the integration of the island into the United Kingdom, which reached the apogee of its imperial and economic power later in the century. After the Act of Union of 1801, there was ongoing resistance to the new constitutional arrangements as well as to other sociopolitical realities, and the relationship between the two islands was often fraught. At a political level, the Westminster government’s responses were dominated by the use of force, along with the suspension of rights enjoyed by those living elsewhere in the United Kingdom, through the passing of legislation known as the Coercion Acts. Farrell (1986) calculated that 105 such pieces of legislation were passed in Westminster between 1801 and 1921. The unequal relationship between Britain and Ireland and the repressive approach to the governance of the latter was legitimized through the use of racialized narratives of difference that implied that the native Irish were incapable of ruling themselves. This is well illustrated in the following 1849 quotation from English historian Henry White (1812–1880):

Thus we see how the United Kingdom is divided between two totally distinct races, the one of Gothic and the other of Celtic origin. Undoubtedly everything great that has been accomplished for several hundred years in this country has been done by the people of Gothic race—by the Saxons of England and Ireland, and the Lowlanders of Scotland. Literature, arts, commerce, industry, civilisation, have all been the work of their hands. We must not, however, infer that the Celts are a permanently inferior race. It would be fair to suppose that ... their position has been depressed by peculiar circumstances in their history, and to hope that at some future day they may rise to the level of their neighbours. (White 1849:77)

White, who was educated at the universities of Cambridge and Heidelberg, expressed an attitude towards the Irish “Celts” that was consistent with the attitudes held by much of the establishment in Victorian England (Curtis 1968; Lebow 1976; Lengel 2002; Monacelli 2010). At the time of White’s publication, the people of Ireland were suffering an immense subsistence crisis that was later deemed among the worst in human history (Ó Gráda 2007). The Great Irish Famine, between 1845 and 1852, resulted in nearly one million deaths: one-eighth of the total population of the island (Boyle and Ó Gráda 1986). The background, course, and outcome of this calamity and, in particular, the role of the British government, remain controversial to this day (Whelan 2004). Most of this controversy lies in the complex historical relationship between Ireland and England, which also determined social policies toward the poor and the marginalized in 19th-century Ireland.

**Fig. 1:**
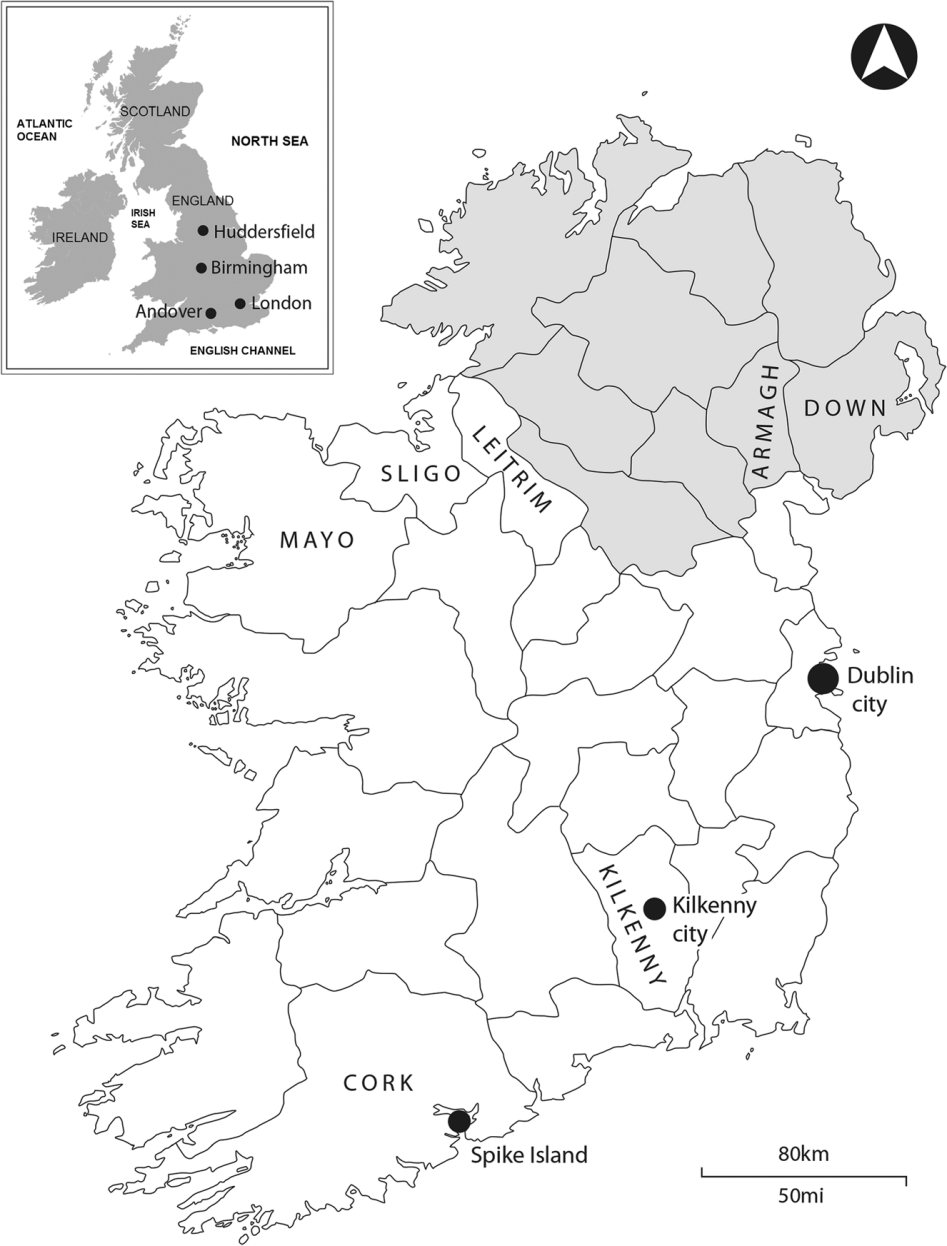
Map of Ireland showing Kilkenny City, Spike Island, and the counties and other locations mentioned in this article. The province of Ulster is shaded in gray. (Map by Nick Hogan, 2017.)

This study uses bioarchaeological approaches to explore Victorian characterizations of the bodies of Irish subjects. The study is based on the analysis of two significant skeletal samples excavated in recent years: the famine cemetery at the Kilkenny Union Workhouse, used between 1847 and 1851 (Geber 2015), and the convict cemetery at Spike Island, county Cork, dating from ca. 1860 to 1883 (Barra O’Donnabhain 2019, pers. comm.) (Fig. 1). Both include interments that represent the poorer cohorts of the Irish population during the mid-Victorian era. The sociopolitical context in which they lived determined their lives, as well as the manner in which they were confined and treated after death. Through the analysis of their skeletons, it is possible to gain valuable insight into the living conditions of these people and to view their lives from a perspective that is not provided by historical sources (Geber 2014).

## Ireland in the Nineteenth Century: Poverty, Famine, and the Social Context of the Act of Union

Though ruled indirectly from London since the Middle Ages, Ireland only became a constituent part of the United Kingdom with the passing of the Act of Union in 1801. The Irish parliament in Dublin, which had existed in some form since the end of the 13th century (Lydon 1997), was abolished. Ireland was thereafter governed directly from Westminster. The background to the union was complex, and the political rationale for constitutional unity between Ireland and Britain had arisen from periods of social unrest and a rising fear of a French invasion (Beckett 1981; Connolly 2000). Economically, the union was supposed to mean that Irish merchants gained greater access to a wider market. In practice, the union did not prevent the further geopolitical marginalization of Ireland (Larkin 2014). The island remained impoverished and poorly developed compared to its eastern neighbor. Unfavorable trade rules ensured that it lagged behind in terms of the rates of industrialization and economic development (Geary 1995). Access to land ownership was highly inequitable and strongly biased toward the often absentee Anglo-Irish ascendancy, most of whom were descendants of 17th-century Protestant settlers from England, Henry White’s “Saxons of ... Ireland” (White 1849:77). Tenant farmers were accorded much less favorable conditions than their British counterparts (Smith 1993). As a result, large landed estates in Ireland were often poorly managed and little investment was made to develop the rural economy. These factors, in combination with a population increase in part driven by the introduction of the potato in the 17th century, resulted in the deterioration of social conditions for the poorest cohort of Irish society in the centuries prior to the Great Famine.

There are numerous accounts of the extreme levels of poverty endured by the poor in Ireland in the lead-up to the famine (Plumptre 1817; Stanley 1833; Inglis 1835; Kohl 1844; Nicholson 1847; de Tocqueville 1997; de Beaumont 2006). Continental European visitors were perplexed as to how this could occur in one of Europe’s wealthiest states. In the decades prior to German unification, for example, German visitors to the United Kingdom looked to Britain, with its prosperity and relatively liberal constitutional monarchy, as a possible role model. However, they were appalled by the extremes of wealth and poverty they encountered in Ireland. One such visitor, Baroness Magdalena von Dobeneck (1808–1891) wrote in 1832: “What a difference there is between England and Ireland! What miserable hovels! ... Here an opulent manor house with proud avenues, there stony land that only with effort yields potatoes!” (Bourke 2011:179–180). Living conditions of the poor were indeed notoriously bad. At the time of the 1841 census, the worst grade of accommodation (Class 4) was home for nearly half of all families in Ireland (Census of Ireland Commission 1843:xiv–vi). This type of housing comprised a mud cabin consisting of only one room or, as was mainly the case in the towns and cities, a larger overcrowded house inhabited by up to five families. One early 19th-century account described how the poor were constantly exposed to cold and humid conditions, often with nothing more than a damp clay floor on which to sleep (Tighe 1802:480). An Irish account from 1843 stated that it was

impossible not to mourn over the general aspect of the cottages. The tent of the Red Indian and the hut of the Esquimaux, are constructed with a greater degree of care and more attention to their rude notions of comfort, than the cabin an Irish peasant erects on the side of the road, or mountain. (S. Hall and A. Hall 1843:290)

Social class was also distinctly expressed in terms of diet and food access. From the mid-17th century onward, the potato crop became, more or less, the only crop on which the vast majority of the poorest members of society subsisted (Feehan 2012). The potato thrived in the Irish climate and was, together with milk that was also a staple of the poor, a relatively good source of nutrition, vitamins, and calories (Crawford 1988). The monoculture economy generally resulted in annual periods of food scarcity and even starvation at the beginning of each summer, when the old crop had been exhausted and before the new produce could be harvested (Sexton 2012). The social and other consequences of this more or less annual occurrence of hunger and deprivation among the poorest of the population paled in comparison to the catastrophe that resulted from the potato blight that first appeared in Europe in 1844. This windblown disease of the potato crop originated in the New World and reached Ireland in August 1845, resulting in the notorious Great Irish Famine (An Gorta Mór in the Irish language), which lasted until the early 1850s. The plant disease spread quickly, infesting potato fields and destroying crops across all of Ireland in as little as a month (Feehan 2012). The situation was exacerbated by failed government policies that attempted to combat the natural disaster with laissez faire and minimalist market-intervention policies. About one million people are estimated to have died from starvation-induced conditions in Ireland between 1845 and 1852 (Boyle and Ó Gráda 1986), while the long-term impact included a hemorrhage of emigration from the island that was not reversed for over a century. The failure of the potato crop occurred across Europe in the late 1840s, but did not result in similar rates of mortality. The devastating impact in Ireland was due to the particular socioeconomic conditions that prevailed on the island and made it especially vulnerable to the failure of a single crop.

## Controlling the People: Perceptions of Race and Class

Throughout the 19th century, the Irish were increasingly caricatured in the British and American press as morally and socially inferior. These caricatures were based on the conception of the Irish Celts as being fundamentally different from the English or American Anglo-Saxons (O’Donnabhain 2000; de Nie 2004). While this perception of Irish inferiority was rooted in the idea of race, it also had a strong class dimension that was in keeping with broader perceptions in Victorian society of the poor being “a race apart” (Stocking 1987; Orser 2011). This is evident when contemplating how the concept of Irishness was perceived within Ireland at the time. Finnegan (2014) has discussed how Abraham Hume (1814–1884) and John McElheran (d. 1859) —Irish ethnologists from upper-class backgrounds—attributed “racial” differentiations in Ireland to class and religion, rather than ancestry. The link between religion and social inferiority dated back to the Reformation and the subsequent replacement of the old elites by a new Protestant ruling class. In 1803, just shortly after the Act of Union, an anonymous former member of the Irish Parliament gave the following description of the “lowest class of the Irish”: “They are certainly, for the most part, thievish, lawless, dishonest and destitute of a sense of equity. (The people of the greater part of the province of Ulster are not meant to be included in the whole of this censure.)” (C. & R. Baldwin 1803:48). The sectarian implications of this statement would have been clear to any contemporary reader, who would have known that the greatest concentration of Protestants was in the province of Ulster. What the author of this quote defined as “lower class” is unclear, but he may have included anyone from the level of small tenant farmers and tradesmen to cottiers, day laborers, servants, and, finally, the destitute (Keenan 2000). Poverty was viewed as a moral failing on the part of the poor, and, in order to control this cohort of the population, moral reforms were proposed by politicians as part of broader social reforms.

This denigration of the population of Ireland in general, and the Irish poor in particular, did not go uncontested (Romani 2016). The idea that there were inherent biological differences between the English and the Irish “races” was challenged at the time, although this was a minority opinion and one that was still argued from an evolutionary perspective (Babington 1895). Most of the contestation came from within Ireland. An example of this is the 1899 publication by the Irish-language scholar and historian Edmund Hogan (1831–1917) entitled *The Irish People: Their Height, Form, and Strength*. It was a compilation of various positive and flattering accounts of the Irish that he produced in order to defend his own people “against shameless and systematic calumny.” He aimed to prove that they were not like “Hottentots” or the “veriest savages on the face of the earth.” Nor were they “like baboons” or the savage caricatures in *Punch*, or “the lowest races of Australia” (Hogan 1899:11–12). The negative characterizations of Africans and Australian aboriginals that are implicit in Hogan’s work reveal the deeply engrained nature of the hierarchical concept of race and the insidious way in which this perspective had been internalized, even by those who were themselves portrayed as inferior.

Indeed, Hogan betrays how those so characterized found themselves trapped within the discourse of race as they struggled to affirm their own place among the “civilized.” It is not surprising, then, that there were opinions expressed within Irish émigré communities in the U.S.A. before and during the American Civil War (1861–1865) that were anti-abolition, and that they even argued for the expansion of slavery into the Northern states. Such calls were most likely driven by anxieties among the Irish American community about their low social standing, which found expression in attempts to distance themselves from those perceived to be lower on the social ladder (Osofsky 1975; Shannon 1989:54–56; Ignatiev 1995; Kenny 2003).

## Burials of the Poor and the Marginalized: The Interments at the Kilkenny Union Workhouse and Spike Island Prison

Research into the bioarchaeology of 19th-century Ireland is a relatively recent phenomenon. While historical archaeology—termed “post-medieval archaeology” in Ireland—has expanded significantly in recent decades (Horning et al. 2007), the study of human remains in Ireland has had a predominant focus on the prehistoric and medieval periods (O’Donnabhain and Murphy 2014). A primary reason for this is that the datasets for much bioarchaeological research in Ireland during recent decades have derived from development-driven rescue archaeology. Excavations at postmedieval cemeteries are rare, as most of these are still in use. Despite this, the significant scientific and historical value of 19th-century graveyards in Ireland has been made evident in recent years, both through rescue and research excavations.

**Fig. 2:**
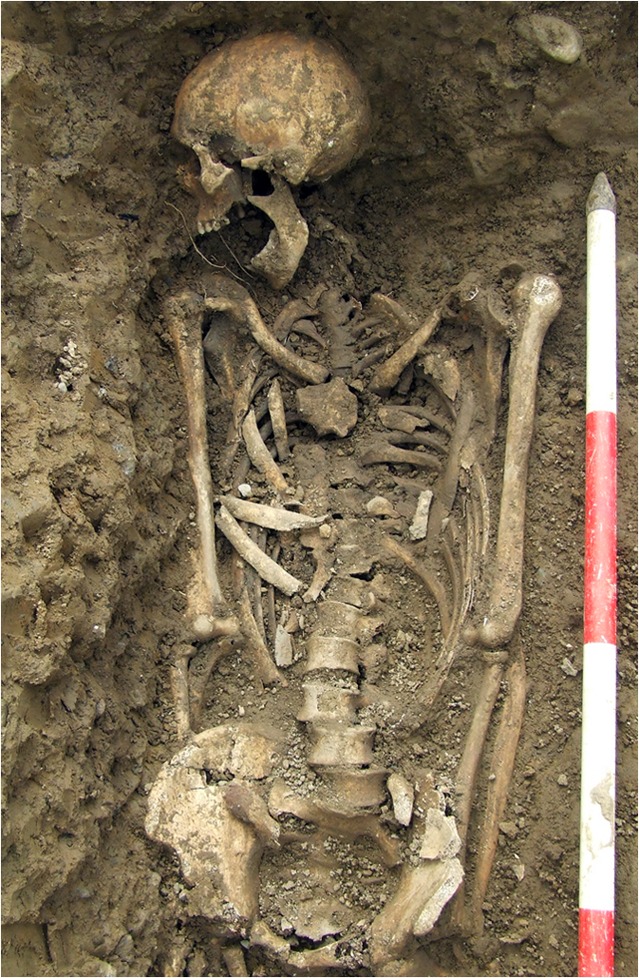
An adult male skeleton (Burial No. DCCXLIV) in situ, from one of the mass burials at the Kilkenny Union Workhouse. He is estimated to have been between 36 and 45 years of age at the time of death. He was approximately 174 cm tall, and his dentition had clay-pipe facets on the anterior teeth. His remains showed no evidence of skeletal trauma. (Photo courtesy of Margaret Gowen & Co. Ltd., 2006.)

In 2005, human remains were encountered during an archaeological evaluation of the grounds of the former workhouse in Kilkenny City. An archaeological excavation was undertaken the following year and revealed what is currently one of the largest archaeologically investigated mass-burial grounds in the world. A total of 63 neatly arranged burial pits containing the skeletal remains of at least 970 individuals were exposed (Fig. 2). The dead had been interred in simple pine coffins that had been stacked on top of each other, with an average of four to five layers per pit. More than half of all those buried (53.8%) were children aged less than 15 years at the times of their deaths. Of those over the age of 15 years at the time of death, 216 could be identified as males and 200 as females (Geber 2015, 2016). While many of the skeletons were highly fragmented, the trabecular and cortical bone was generally in a good state of preservation.

Subsequent historical research has confirmed that these burials relate to a peak in mortality that occurred between August 1847 and March 1851, at the height of the Great Famine. The Kilkenny workhouse was one of 163 such institutions that were opened all over Ireland between 1841 and 1853 following the passing of the Irish Poor Law Act of 1838. The Poor Law was a response by the government in London to the dire social conditions in Ireland. The reform was based on the Poor Law Amendment Act (Eyre and Spottiswoode 1899) for England and Wales that had been passed by the Whig government (1830–1834) four years prior to the extension of the system to Ireland. A fundamental aspect of the Irish Poor Law Act was that state aid (in terms of food and accommodation) would only be provided on the basis of utmost necessity and in exchange for physical labor in a workhouse.

**Fig. 3:**
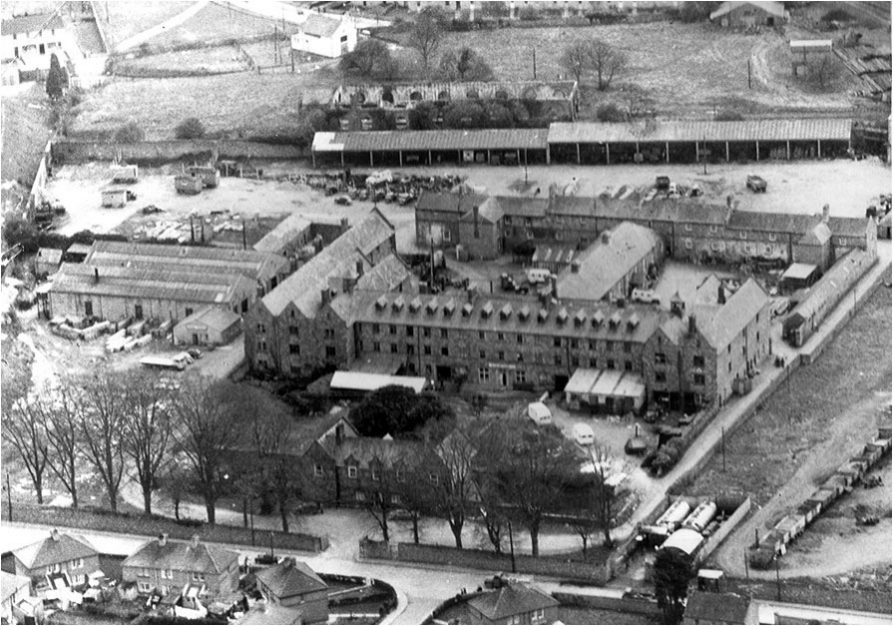
Aerial photograph (ca. early 1960s) of the Kilkenny Union Workhouse. The mass burial ground was located in the northeast corner of the boundary wall, visible in the *upper-left corner* of the photograph. (Photo courtesy of Karen Deegan and the Kilkenny County Library.)

The Irish workhouses were almost all constructed following an identical design, comprising a massive H-shaped accommodation and infirmary block located behind an administrative building and enclosed by 8 ft. high stone walls (Raftery 1995). Unions served as the administrative units of the Poor Law system. The Kilkenny Union Workhouse, with capacity for 1,300 inmates, was opened on 21 April 1842 (Fig. 3). On that first day of admission, 30 applications were accepted (Geber 2015). During the Great Famine, however, over 4,300 people were housed in the facility and in auxiliary premises rented nearby, so that the institution became grossly overcrowded, with mass deaths occurring indirectly as a result (Geber 2015, 2016).

**Fig. 4:**
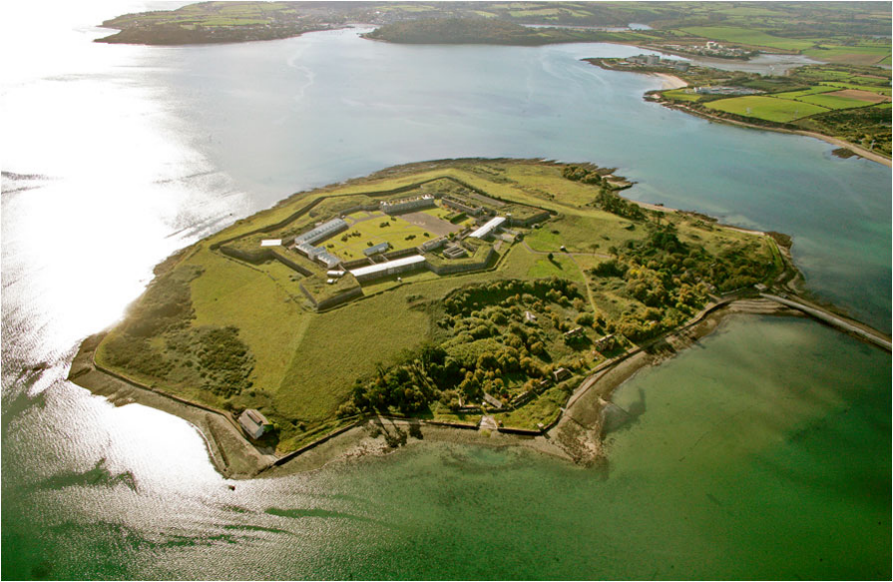
Aerial photograph of Spike Island, County Cork, viewed from the northeast, showing the 19th-century fortifications that were completed using convict labor. The original convict cemetery was buried ca. 1860 under the sloping glacis in the *foreground*, and a second convict burial ground was then established at the southwest side of the island, at the *top* of this image. (Photo courtesy of Con Brogan, National Monuments Service, 2013.)

The male convict depot on Spike Island in Cork Harbor received its first prisoners in October 1847 and operated for the following 36 years (McCarthy and O’Donnabhain 2016). The first prisoners found themselves in a large Napoleonic-era fortress that had been left unfinished after the defeat of the French at Waterloo in 1815 (Fig. 4). Using the fort as a prison served the dual purpose of easing famine-related overcrowding in the county and city jails throughout the island of Ireland. It also provided an unpaid labor force to complete the fortifications on Spike Island. While the early Victorian approach to incarceration preferred the isolation of prisoners in single cells, this was not possible on Spike Island, where convicts were held in overcrowded dormitories for the first two decades of the prison’s operation. Initially planned to hold just a few hundred convicts, by 1850 the island housed over 2,300 men, making it the largest prison in the United Kingdom, as it was then constituted.

**Fig. 5:**
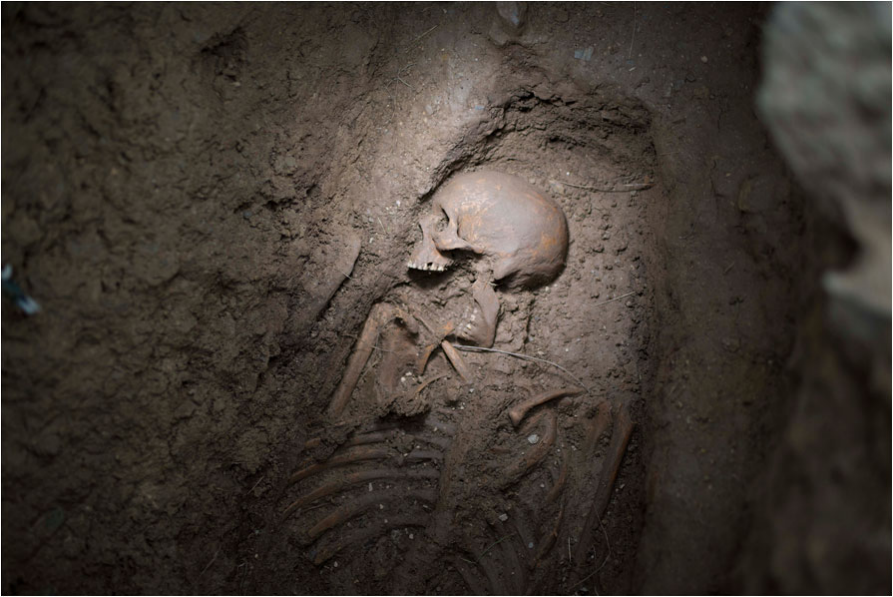
One of the Spike Island convict burials (Burial No. C.318) in situ. This was a younger adult male who was approximately 174 cm in stature. He was a smoker and preferred to hold his pipe on the right side of his mouth. He had incurred a broken nose at some stage of his life. He was buried in a coffin, the outline of which was detectable at the time of excavation. (Photo by Stephen Bean, 2013.)

**Fig. 6:**
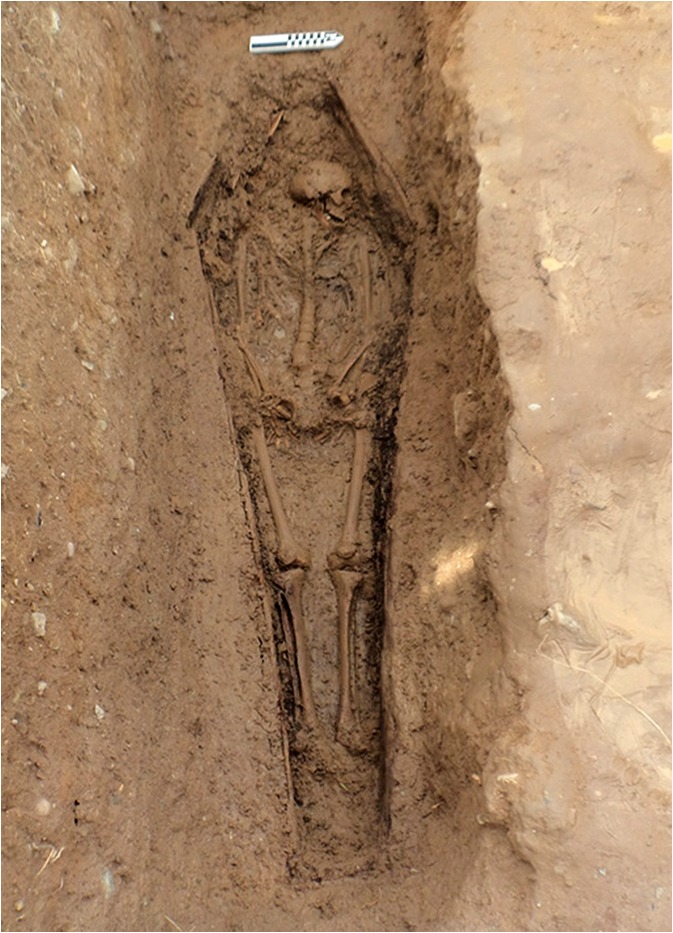
An in situ, older adult male from one of the Spike Island convict burials (C.507). He was approximately 170 cm tall and a smoker who preferred to hold his pipe on the right side of his mouth. He was buried in a coffin, the timbers of which survived. There was no evidence of trauma in the skeleton, but the presence of periostitis on the pleural surface of three unsided rib fragments indicate that he suffered from some form of pulmonary disease, probably tuberculosis. (Photo by Barra O’Donnabhain, 2014.)

The overcrowded conditions contributed to a very high mortality rate, peaking at 286 deaths (>12% of the inmates) in 1853. A royal commission established that year recommended reducing prisoner numbers on the island to below 1,000. This had a dramatic effect, and, from 1860 to 1883, prisoner deaths averaged at 6.3 per annum. A total of just under 1,200 men are recorded as having died in the prison between 1847 and 1883. Over 80% of these deaths occurred between 1850 and 1854, and were interred in a cemetery on the east side of the island. Between 1860 and 1862, that cemetery was buried under up to 6 m of fill during the final construction phase of the fortifications. A new convict graveyard was established at the western end of the island and contains the remains of about 150 men. Excavations were carried out in this cemetery from 2013 to 2018 as part of the Spike Island Archaeological Project and revealed a regimented series of graves that were mostly of uniform depth and spacing. Twenty-six graves were excavated between 2014 and 2016, and, in all cases, the convicts were buried in coffins (Figs. 5, 6). The burials date from ca. 1860 to 1883 and postdate the Great Famine and the period of highest mortality in the island prison. However, it is likely that most, if not all, of the individuals whose skeletons were uncovered had lived through the famine, and some are likely to have witnessed the gross overcrowding of the prison (Barra O’Donnabhain 2019, pers. comm.).

For both the Kilkenny and Spike Island assemblages,^1^ osteological analysis of the human remains was based on standard methodological praxis (Buikstra and Ubelaker 1994). Age at death was estimated from dental mineralization, tooth eruption, and epiphyseal fusion in subadults (Scheuer and Black 2000), and from morphological changes to the pubic symphyses and auricular surfaces of the os coxae, sternal end of the ribs (İşcan et al. 1984; Lovejoy et al. 1985; İşcan and Loth 1986; Brooks and Suchey 1990), and cranial suture obliteration (Meindl and Lovejoy 1985) in adults. Sex for adult individuals was estimated from cranial and pelvic morphological traits (Sjøvold 1988; Mays and Cox 2000). Living stature was estimated using equations formulated by Trotter and Gleser (1952, 1958). Only adult individuals (>18 years) were considered for this study.

## Recovering Identities: A Bioarchaeology of Social Marginalization in Nineteenth-Century Ireland

A distinctive mode of dress, tobacco use, and a readiness for violence became part of the stereotype of the Irish man. Edmund Hogan referenced these traits in his summary of the intentionally demeaning caricaturing of the Irish in the British press:

They took as their model the very lowest type of the Englishman, adorned him with a tattered coat, kneebreeches, a battered hat, a clay pipe, a shillelah [a wooden walking stick and a weapon, also spelled shillelagh, see below], and presented him as the typical native of Ireland, The Predominant Partner, of course, made endless fun of such a droll figure, and while devoting himself to the complacent survey of his own immaculative and irreproachable person, prayed thus within himself: “O God, I give Thee thanks, that I am not as the Hirishman.” (Hogan 1899:7)

Hogan astutely noted that the manner in which the Irish poor were despised was mirrored in a lack of respect for the poor across the United Kingdom, irrespective of nationality. Systematic and demeaning treatment of the poor in England certainly occurred. One notorious case was the Andover (Hampshire) workhouse scandal in 1845 (Longmate 2003), where the inmates were denied their food rations and resorted to eating garbage. Another infamous case was the filthy conditions and extreme neglect that workhouse inmates in Huddersfield (West Yorkshire) had to endure during a typhus outbreak in 1848. A shocking report of the conditions, which were deemed to be worse than those in Andover, was first published by the *Leeds Mercury* newspaper (*Leeds Mercury*^1848^), and it resulted in a nationwide disgust and outrage amongst the public (Fowler 2008). Even Friedrich Engels (1845), a champion of the poor who was one of the first to highlight the link between people’s material conditions and health, wrote of the English urban working class as a race apart, who were physically degenerate, robbed of all humanity, and reduced both morally and intellectually to near-bestial condition. The impoverished Irish who had immigrated to England during this time would, nevertheless, still find themselves being perceived as socially inferior to the “native poor.” There was concern at a government level that the “turbulent and irregular habits” of the Irish would negatively influence the morals of the poorer populace of Great Britain (Cornewall Lewis 1835). Friedrich Engels (1845) was of a similar view in the 1840s, suggesting that the English urban proletariat had been reduced to their degenerate state as a result of the dehumanizing effects of economic exploitation, but also by competition and association with the coarse, dissolute, volatile, and drunken Irish. It should be noted, however, there were also those of the opposing opinion who stated that Irish men and women immigrants were “efficient workmen” with “highly favourable” moral conduct (Cornewall Lewis 1835; Nicholls 1856:183). According to census records, in 1841 there were 415,725 Irish-born people residing in England, Wales, and Scotland. Twenty years later, and largely due to mass emigration during the Great Famine, this number had almost doubled, to 805,717, which corresponded to 3.6% of the total population of Great Britain (MacRaild 2011:table 2.1).

### “Here We Find No Trace of Degraded Dwarfs”: Irish Stature in the Nineteenth Century

In December 1836, an anonymous writer published an article entitled “Attractions of Ireland” in the *Dublin University Magazine*. The article described the condition of Ireland and the Irish people, and was rhetorically anti-British in its tone. Despite this, the article contained negative stereotyping of the Irish poor, reflecting the often conflicted and ambivalent nature of the identity of the Anglo-Irish ruling class (Dublin University was a bastion of the elite). One of the writer’s main contentions was that the Plantation of Ulster, where Scottish Protestants were settled in the 17th century at the expense of the local Roman Catholic population (Robinson 2000; Bardon 2011), had forced the “native Irish” from counties Armagh and Down to migrate westward to counties Mayo, Sligo, and Leitrim, where they, through “the worst effects of hunger and ignorance,” had degenerated both physically and mentally. He wrote how the descendants of these displaced peoples were “a wide-mouthed, flat-nosed, low-browed, and hollow-eyed, rabble, poor in person and pitiable in intellect,” and that their faces “bear barbarism on their very front” (*Dublin University Magazine*^1836^:667). Furthermore, this degenerated people was described as “five feet two upon an average, pot-bellied, bow-legged, abortively-featured” that “fright the sister island with annual apparitions of Irish ugliness and Irish want” (*Dublin University Magazine*^1836^:669). Sixty-three years later, Edmund Hogan took great offense at this statement, and through the numerous accounts combined in his book (see above) he was able to affirm that, amongst the peasantry of Ireland, “[h]ere we find no trace of degraded dwarfs” (Hogan 1899:123).

Height as a physical characteristic of people and “race” was of particular interest to 19th-century anthropology, principally because of the perceived strategic advantage to the military of having tall, robust soldiers. Stature is still a measurement frequently discussed in anthropometric studies today, but now as a reflection of health and living conditions. It is habitually used by economic historians and others as a proxy for assessing standards of living in human societies (Steckel 1995; Carson 2005; Young et al. 2008). Disparities in height between population groups are generally interpreted as reflections of sociocultural and socioeconomic divisions (Rosenbaum 1988). Floud, Wachter, and Gregory identified four main non-genetic factors determining terminal stature: nutritional status, social and geographical inequalities, urbanization and disease environment, and rising or falling real wages (Floud et al. 1990). Terminal stature from skeletal remains is also frequently adopted as a measure of biocultural adaptations and health by bioarchaeologists (A. Goodman and Martin 2002; Mummert et al. 2011; Vercellotti et al. 2011). However, it has not been common in the discipline to explore links between stature in assemblages and causative factors, such as those proposed by Floud et al. (1990), although there are some notable exceptions (Boldsen 1993; Sellevold 1993; Vercellotti et al. 2011; Arcini et al. 2014).

In studies of the 19th century in other disciplines, there has been a significant focus on secular changes as a result of social consequences, with average-height reductions following increasing levels of urbanization and industrialization (Baten and Murray 2000; de Beer 2010). In the Irish context, the focus has been on historical conscript anthropometric data gathered by the British military, which have indicated that Irish recruits were taller than their English counterparts by an average of ½–1 in. (12.7–25.4 mm) (Floud et al. 1990). Mokyr and Ó Gráda have discussed this difference as a potential reflection of the Irish reliance on a potato diet (Ó Gráda 1991; Mokyr and Ó Gráda 1994), which was relatively nutritious (Crawford 1988) compared to the diet of the English working class, which was dominated by bread and tea (Wohl 1983). Considering the substantial differences between Irish and English societies in both industrialization and urbanization, a disparity in adult statures between these two populations is a trend that might be expected. The bioarchaeological evidence, however, suggests that this may not have been the case.

The estimated living stature of adult females at the Kilkenny Union Workhouse averaged 158.2 cm, while the mean height of males was estimated at 171.4 cm (Geber 2015). At Spike Island, the average height of a sample (*n*=17) of the men interred in the burial ground was estimated at 167.4 cm. The Kilkenny estimates are higher compared to anthropometrical data of Irish population samples in the 19th-century, while the Spike Island estimates are similar to those reported means (Table 1). Anthropometric data that are available from the Spike Island prison archival records indicate that the average stature of males incarcerated during 1849–1850 was slightly less than that provided by skeletal estimates, however, a direct comparison between osteometric and anthropometric data is not possible due to methodological issues.^2^

**Table 1:** Estimated and recorded statures of 19th-century, living Irish males

Sample	*N*	Height Data	Height ($\overline{x}$)	Source
Kilkenny Union Workhouse (paupers), all ages, 1847–1851	186	Osteometric	171.4 cm (67.5 in.)	Geber 2015
Spike Island Prison (convicts), all ages, ca.1860–1883	17	Osteometric	167.4 cm (65.9 in.)	Barra O’Donnabhain 2019, pers. comm.
East India Company (recruits), all ages, 1800–1809	432	Anthropometric	164.9 cm (64.9 in.)	Mokyr and Ó Gráda 1994
East India Company (recruits), all ages, 1810–1814	1,350	Anthropometric	166.1 cm (65.4 in.)	Mokyr and Ó Gráda 1994
New South Wales (convicts), all ages, 1817–1840	5,005	Anthropometric	167.7 cm (66.0 in.)	Nicholas and Steckel 1991
Lower Canada (convicts), all ages, 1820s	809	Anthropometric	169.7 cm (66.8 in.)	Morin et al. 2016
Kilmainham Prison (convicts), >23 years, 1840s	1,400	Anthropometric	168.4 cm (66.3 in.)	Ó Gráda 1996
Clonmel Prison (convicts), 25–29 years, 1845–1849	521	Anthropometric	168.6 cm (66.4 in.)	Ó Gráda 1996
Spike Island Prison (convicts), all ages, 1849–1850	529	Anthropometric	166.1 cm (65.4 in)	Barra O’Donnabhain 2019, pers. comm.
Perth General Prison, Scotland (convicts), 23–50 years, 1860s	110	Anthropometric	169.3 cm (66.7 in.)	Beddoe 1870
British Army (recruits), 23–50 years, 1860s	1,517	Anthropometric	170.8 cm (67.3 in.)	Beddoe 1870

To investigate whether there was a height discrepancy between Irish and English populations during the 19th century, femoral lengths (as a proxy for stature) from the Kilkenny Union Workhouse and Spike Island samples were compared with 19th-century human skeletal samples (*n*=263) from London (Wellcome Osteological Research Database 2016). The latter samples derive from the lower social-status burial grounds of St. Bride Lower on Farringdon Street and Crossbones in Southwark, and also Chelsea Old Church in Chelsea, which was used by the middle to upper social classes (Table 2).

**Table 2:** Femur bone lengths (mm) from Irish and English skeletal samples

Sample	Males					Females				
	*N*	Min.	Max.	Mean	SD	*N*	Min.	Max.	Mean	SD
Kilkenny Union Workhouse, 1847–1851	133	388.0	507.0	455.0	24.4	124	372.5	475.0	419.5	21.3
Spike Island Prison, ca. 1860–1883	17	410.0	502.0	445.5	19.2	––	––	––	––	––
London, England, 18th–19th centuries^1^	147	395.0	513.0	451.6	24.9	116	355.0	468.0	421.0	22.1

^1^This sample comprises collated data from the following postmedieval cemeteries: St. Bride’s Lower (1770–1849), Chelsea Old Church (18th–19th centuries), and Crossbones (1800–1853) (Wellcome Osteological Research Database 2016).

Using a t-test to investigate dissimilarities, the comparison failed to detect any significant differences in bone lengths for either males (*t=*1.169, *df=*278, *p=*0.244) or females (*t*=-0.516, *df*=238, *p*=0.607). The variance between the Irish and London samples was also surprisingly similar, as indicated from a Levene’s test for both males (*F*=0.001, *p*=0.974) and females (*F=*0.159, *p=*0.690). Neither was there any significant difference in mean when comparing the femoral robusticity values (as a proxy for biomechanical loading) between the Irish and English samples (males: *t*=-0.433, *df*=256, *p*=0.665; females: *t*=0.634, *df*=238, *p*=0.527).

In 1870, the British anthropologist and ethnologist John Beddoe (1826–1911) published the book *Stature and Bulk of Man in the British Isles* in which he discussed variations in height and weight of males across the United Kingdom as it was then constituted. Beddoe was a polygenist, and his work was highly influenced by the prevailing late 19th-century discourse of “race.” In fact, he was one of the main proselytizers of this idea in his time (Beddoe 1885). In a paper delivered in June 1870 at the Anthropological Society of London entitled *The Kelts of Ireland*, Beddoe discussed how he had developed an “Index of Nigrescence” from which he concluded that the Irish were of mixed race (Morash 1998). Despite his preoccupation with the “relations of stature to race,” Beddoe provided some interesting observations on the influence of non-racial parameters when he argued that variance in stature was influenced by levels of urbanization, diet, and socioeconomic factors. One of Beddoe’s main conclusions was that Irish recruits were “almost equal in stature and fall somewhat below in weight” compared to English and Scottish soldiers (Beddoe 1870:186–191). Considering that the majority of the measured Irish male recruits in his study would have been born before or during the Great Famine, his conclusion is interesting when discussing the value of using stature as a proxy for health and living conditions in 19th-century populations.

There is no exclusive factor influencing terminal human stature, but rather an intricate variety of causes and circumstances. This is apparent from the Irish evidence of both osteometric and anthropometric data from the 19th century (for a critical discussion on the use and interpretation of Irish anthropometric data, see Ó Gráda [1996]). The social changes that Ireland underwent during this period were substantial (Boyce 2005), and they were not linked only to the Act of Union in 1801 and the Great Famine. These changes also occurred as a result of reforms, such as the Poor Law Act of 1838 (see above); the Disestablishment Act in 1869, which separated the established Anglican church (Church of Ireland) from the state (Beckett 1981:364–369); and the Land Acts of 1870–1903 that facilitated the transfer of land ownership from landlord to tenant (Solow 1971; Beckett 1981:389–394). What is evident from the current data is that, despite these social changes and the socioeconomic and cultural differences between the two societies, the Irish from these two assemblages were not impaired in stature compared to the English reference samples.

A study of stature and its relation to socioeconomic status in post-famine Ireland by Young et al. (2008) observed statistically significant relationships between terminal height and occupation, education, and migration status. More surprisingly, however, the same study concluded that an increase in average stature of males was the greatest in the counties of Ireland that had been most affected by the famine. While Young and coworkers interpreted this trend as a reflection of a relative increase in living standards due to diminishing competition for local resources following the substantial population loss, it also further highlights the difficulties in making assumptions based on stature if historical and cultural contexts are not taken into consideration (Relethford 1995).

### The Fighting Irish? Cultural Patterns of Violence

**Fig. 7:**
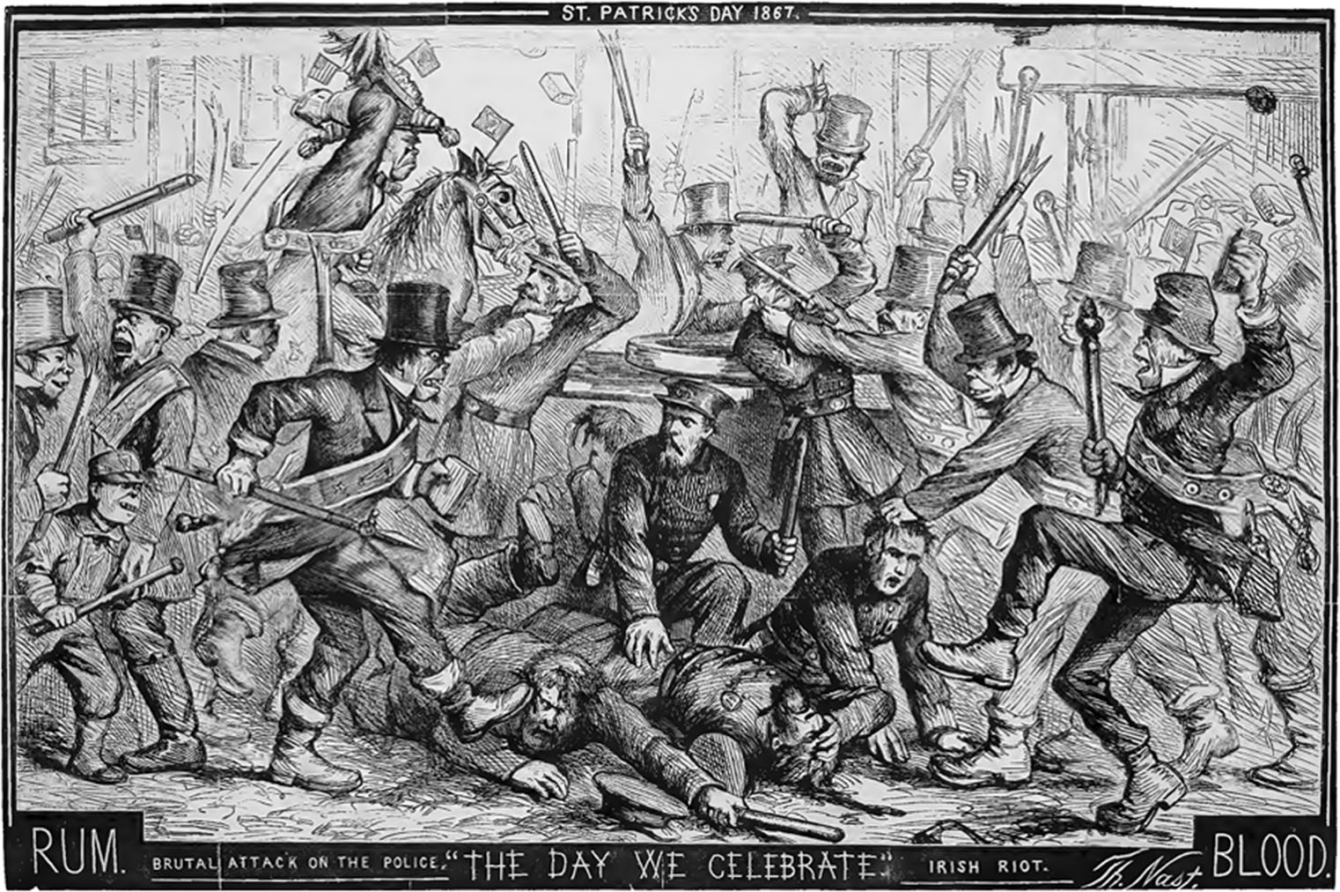
The infamous illustration, *The Day We Celebrate*, by caricaturist and cartoonist Thomas Nast (1840–1902), published in the New York newspaper *Harper’s Weekly* on 6 April 1867, that depicted the Irish in a clear, dehumanizing manner as violent and brutal characters with subhuman features (Nast 1867).

A frequent theme in the stereotyping of the Irish during the 19th century was to ascribe drunkenness and violence to that group. The term “the fighting Irish” has been used since at least the 1830s (Hughes 2006:256). Violence, as a feature of the Irish and Irish culture, was frequently depicted in xenophobic caricatures in the press. This was seen in the British satirical magazine *Punch* (de Nie 2004), as well as American papers, such as *Puck*, *Yankee Notions* (Appel and Appel 1990), and the New York–based political magazine *Harper’s Weekly* (Fig. 7). While modern sociological and psychological studies tend to attribute high rates of violence within communities primarily to socioeconomic factors (Hsieh and Pugh 1993; Fabio et al. 2011), there were also cultural practices associated with the Irish in the 19th century that could explain this perception. These included so-called faction fights, which were not uncommon in rural Ireland during the pre-famine era. Faction fights were a form of ritualized violence that was part of rural folklife. They were often perceived as a form of sport or amusement, and were a means of easing social tensions, even though fatalities could and did occur (O’Rourke 2016). These were usually prearranged occasions, often taking place in at large gatherings, such as markets and fairs (Conley 1999). Faction fights were often based on grounds of identity formed by the places of origin of the antagonists or were perceived as being due to long-lasting family feuds (the reasons for which sometimes had long since been forgotten) (Conley 1999; O’hAodha 2008). Women would occasionally participate in these fights also (O’Donnell 1975; Conley 1995).

Bioarchaeology tracks violence in past societies by assessing the frequency and patterns of trauma, such as dislocations and healed fractures to bone (Lovell 1997; Redfern 2017). Evidence of interpersonal violence is ubiquitous in the bioarchaeological record, and it is clear that cultural practices contribute to determining the patterns of interpersonal violence in archaeological skeletal materials (P. Walker 2001; Novak 2006, 2017; de la Cova 2010). This was highlighted in Brickley and Smith’s (2006) study of a skeletal sample from lower to upper social-class burials at St. Martin-in-the-Bull-Ring from Birmingham, England, dating from the mid-18th to mid-19th centuries. In their study, violence-related injuries (VRIs), which were identified from metacarpal, cranial vault, and maxillofacial fractures, had an 11:1 male to female ratio that highlights a strong link between interpersonal violence and gender. When comparing social class—as determined from burial contexts—there was no significant difference in VRI rates, with the exception of nasal fractures, which were more common in high-status individuals interred in crypts.

The Birmingham sample is contemporary with the Kilkenny and Spike Island assemblages, and thereby provides an opportunity to explore potential sociocultural differences in the patterns of violence between mid-19th-century English and Irish populations. Using the same parameters as those utilized by Brickley and Smith, both similarities and disparities are apparent between the English and the Irish samples (Table 3). In both datasets, males were more likely to sustain VRIs, which suggests that, on both islands, male activities and behaviors left them at greater risk of violence resulting in broken bones. However, the ratio of males to females is substantially lower for the Kilkenny sample compared to Birmingham, particularly in relation to cranial-vault trauma. Overall, cranial-vault blunt-force trauma was present in 6.3% (12/190) of the Kilkenny males and 5.1% (9/175) of the females, a difference which was not statistically significant (χ^2^=0.231, *df=*1, *p=*0.631). This is an interesting observation, as it not only runs contrary to the commonly perceived pattern of violence in human skeletal samples (Novak 2017), but perhaps also reveals aspects of gender relations among the poor in mid-19th-century Ireland. Conley (1995) has discussed how women in late 19th-century rural Ireland clearly acted against the established social norms of the ways they should behave by frequently resorting to violence to solve conflicts. While Conley (1995) related this to the social trauma relating to having experienced the famine, the bioarchaeological evidence from Kilkenny does imply that a gender homology in relation to certain patterns of skeletal trauma was very much in place in the early decades of the 19th century in Ireland.

**Table 3:** Sex-distribution pattern of bone fractures in Kilkenny and Birmingham

Injury	Kilkenny Union Workhouse		St. Martin’s, Birmingham	
	*N* Sexed Individuals (M:F)	M:F Ratio	*N* Sexed Individuals (M:F)	M:F Ratio
All fractures	77:44	1.6:1	75:23	3.7:1
Ribs	24:7	3.4:1	44:7	6.3:1
Metacarpals	13:0	13.0:0	15:1	15.0:1
Maxillofacial	5:1	5.0:1	6:1	6.0:1
Cranial	12:9	1.3:1	5:0	5.0:0
All VRIs	27:10	2.7:1	22:2	11.0:1
Excluding VRIs	50:34	1.5:1	53:22	2.4:1

In the Birmingham sample, 14 of the 16 cases (87.5%) with metacarpal fractures involved the first metacarpal. In Kilkenny, the rate was 61.5% (8/13) for the first metacarpal, followed by 23.1% (3/13) for the fifth metacarpal, 15.4% (2/13) for the second, and 7.7% (1/13) for the third and fourth metacarpals. At Spike Island, two of the male inmates had healed fractures to the first metacarpal (2/3; 66.7%), while another had a fracture of the fifth metacarpal (1/3; 33.3%). Brickley and Smith (2006) interpreted the metacarpal-fracture distribution in the Birmingham sample as reflecting boxing injuries that would have occurred from so-called bareknuckle fights, which became increasingly common in England during the 19th century. If related to interpersonal violence, the fact that there is a lower frequency of first metacarpal fractures in the Kilkenny sample would, in that case, suggest that physical assault using the fists was less common in this population group compared to their contemporaries in Birmingham. This would not necessarily be a bioarchaeological indication that interpersonal violence was less common in Kilkenny, but perhaps, rather, that the cultural expressions of violence differed between the two societies.

Throughout the 19th century and well into the 20th century, stick fighting or *bataireacht*, with the use of a shillelagh, was common in Ireland. Shillelaghs (from the Irish *sail éille*) had a more common use as walking sticks and were traditionally made from a hardwood, such as oak or blackthorn. They had blunt pommels of varying shape that could be used to inflict damage (Hurley 2007). A shillelagh enabled combatants to both attack and defend themselves quickly, with brute and effective force. While stick fighting was not an unknown practice in England, it is believed to have decreased significantly by the early 19th century due to the increased popularity of boxing and prizefighting during this period (P. Walker 1997; Wood 2004). Pierce Egan (1772–1849)—a British sportswriter and journalist of Irish descent—chauvinistically claimed (Egan 1830:13) that, due to their “national character,” the English would only resort to using fists during fights. The Dutch, however, would frequently resort to “the long knife,” while the French and the Germans, according to Egan, would use stones and sticks to “gratify revenge!” Shillelaghs were the weapon of choice during faction fights in Ireland, along with the throwing of stones (O’Rourke 2016). The frequent use of the shillelagh in fights throughout the 19th century in Ireland (Hurley 2007) resulted in this particular style of fighting becoming associated with Irish combatants and is a common theme in their representation (Fig. 7).

**Fig. 8:**
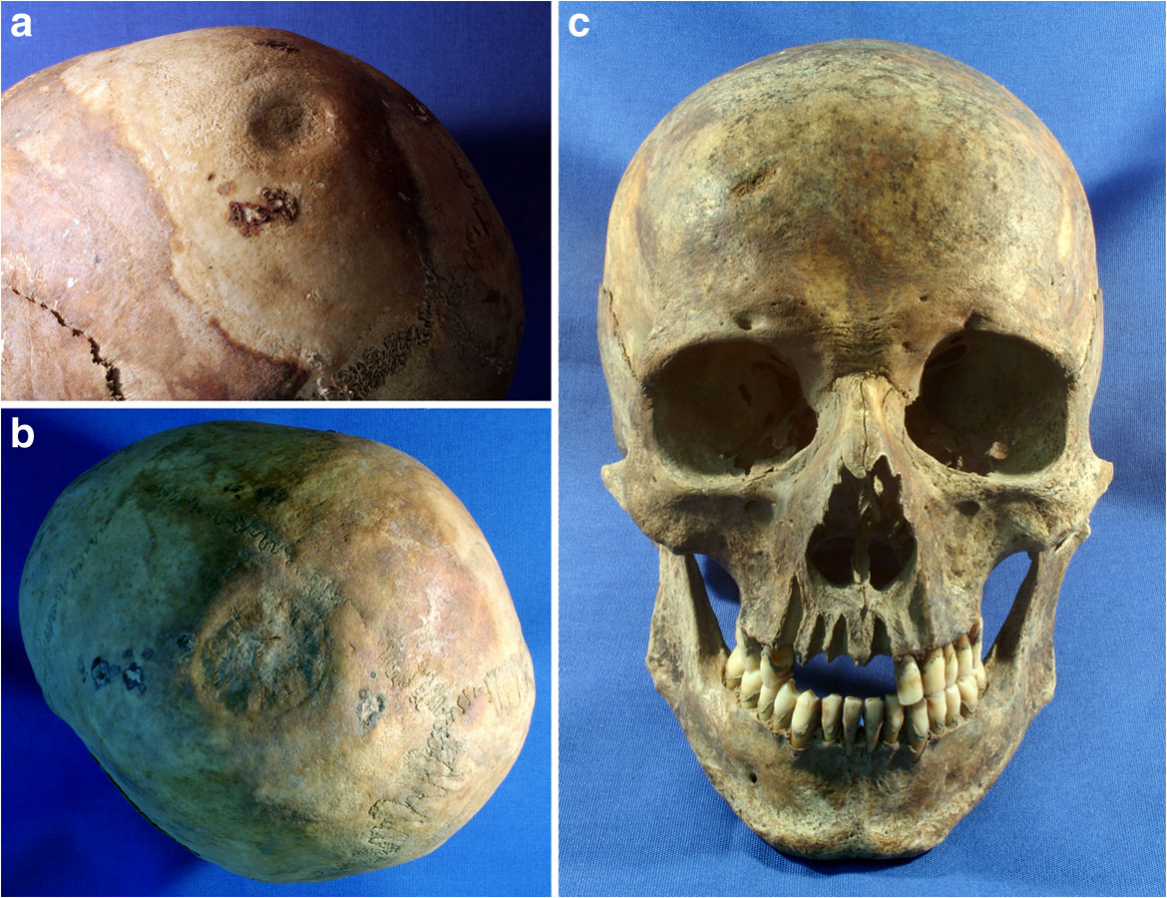
Evidence of cranial blunt-force trauma in three adult individuals from the Kilkenny Union Workhouse mass burials: To the *right*, left parietal bones of an older adult female (*a*=Burial No. CDIV) and middle-adult female (*b*=Burial No. CXVII), and, on the *right*, a portion of the frontal bone of a middle-adult male (*c*=Burial No. CDXXV). (Photos by Jonny Geber, 2009.)

The possible use of shillelaghs or throwing of stones in Kilkenny is evidenced from the frequency of cranial blunt-force trauma (Fig. 8), which was relatively frequent in both males and females (see above). Brickley and Smith (2006) attributed all cranial vault trauma to interpersonal violence. The possibility that some of the lesions noted in Kilkenny could have been related to accidents occurring, for instance, during industrial or agricultural labor, or even during sports, should not be discounted. However, most fractures (17/26; 65.4%) were observed on the left of the skull vault (Table 4), which suggests that an intentional blow from a right-handed aggressor is a likely cause for most of these injuries. The fractures varied in shape, from round indentations that would have been caused by a spherical item to linear and asymmetric depressions that would have been caused by straight-edged or irregularly shaped objects. Stones, or the pommel “hitting” end of a shillelagh, which was made in various shapes and forms (Hurley 2007), could easily have been the cause of some of these. Evidence of blunt-force trauma to the cranial vault was noted in two of the convict burials from Spike Island (2/26; 7.7%). Both injuries were of spherical form and were to the left frontal. Although the small sample size should be noted, this is a similar rate to that noted in the males from Kilkenny, which is not surprising, as the social background of both assemblages is similar.

**Table 4:** Number of cranial fractures in adults from Spike Island and Kilkenny

Bone	Spike Island			Kilkenny Union Workhouse					
	Males			Males			Females		
	Left	Right	?	Left	Right	?	Left	Right	?
*Skull vault*									
Occipital	––	––	––	––	––	2	––	––	––
Frontal	2	1	––	3	2	2	3	1	2
Parietal	––	––	––	6	––	––	3	––	––
Temporal	––	––	––	1	––	––	1	––	––
*Maxillofacial*									
Nasal	2	––	––	3	2	––	––	––	––
Zygomatic	––	––	––	––	––	––	––	––	––
Maxilla	––	––	––	1	1	––	––	––	––
Mandible	––	––	––	––	––	––	––	1	––
TOTAL	4	1	0	14	5	4	7	2	2

When commenting in 1835 on the Irish “moral character,” Lord John Russell, MP (1792–1878), stated in a debate in the House of Commons that in Ireland there “exists, as we unhappily know, a strong propensity to violence and outrage, not merely among a few lawless and ill-regulated persons, but among all, or nearly all, classes of the community” (Russell 1870:402). Russell became prime minister in 1846 and continued to serve as such throughout the Great Famine; he was created Earl Russell for his efforts in 1861. This perception of the Irish as an inherently violent and unruly people was undoubtedly a factor that contributed to the mismanagement and poor judgment shown regarding the relief policies implemented by the British government during the famine period. This recourse to blaming the “character” of the Irish is also apparent in a statement made by the assistant secretary of the treasury, Charles Trevelyan, who affirmed that “the real evil with which we have to contend is not the physical evil of the Famine, but the moral evil of the selfish, perverse and turbulent character of the people” (Woodham-Smith 1962:156). Trevelyan (1807–1886) was in charge of organizing food aid and famine relief in Ireland, and in 1848 he received a knighthood from Queen Victoria (1819–1901) for his “services to Ireland” (Haines 2004).

When contemplating the overall fracture patterns observed in Kilkenny, Spike Island, and Birmingham, and taking into account how violence may have been expressed differently on a cultural basis, there is nothing to suggest from the bioarchaeological evidence that, between these societies, there would have been a substantially different exposure to violence. Many historical narratives of 19th-century Irish society have repeated the claim that it was indeed violent, based on the contemporaneous accounts of faction fights, riots, and rebellions. However, it has also been argued that these accounts were markedly exaggerated (McMahon 2013:1–11). Like Trevelyan and Russell’s rationalizations, such exaggeration served a political purpose in that it paved the way for emergency legislation and the suspension of rights guaranteed elsewhere in the United Kingdom. After the Act of Union in 1801, increases in crime in Ireland were regularly reported during turbulent events, such as the Tithe War of the early 1830s. This was a campaign of mostly nonviolent disobedience by the majority Roman Catholic population against paying tithes to support the minority, but official, state church. Other turbulent events include the campaign to repeal the Act of Union, the Great Famine, and the Young Ireland rebellion of 1848, which was part of the wave of pro-democracy revolutions across Europe that year. In addition to the repressive Coercion Acts, London’s response included the development of state-run prisons, such as that at Spike Island. The Irish penal system was reformed in the aftermath of the Great Famine, and the prison authorities claimed that the success of their innovations could be seen in a gradual decline in criminality as the century progressed, as measured by falling conviction rates and prisoner numbers (Finnane 1997). Others writing at the time linked the decline in the prison population to the amelioration of conditions after the famine and the subsequent rapid demographic decline that was principally due to emigration (McCarthy and O’Donnabhain 2016). The population of the island fell from 8.17 million in 1841 to 4.39 million in 1911.

### Pipe Smoking—Identity and Social Resistance

Descriptions of the laboring classes in Ireland in the 19th century often referred to the smoking of clay pipes (as the stems were prone to breaking, a clay pipe was known colloquially as a “cutty,” a generic word for anything cut short), which eventually became an essential part of caricatures of the Irish peasantry. This was highlighted in an article in the Irish nationalist publication *Duffy’s Hibernian Magazine* in late 1861, that stated that, for the descriptions of Ireland, “[c]aricature, not Truth, is required for the English market,” and that any “caricature of ‘Paddy’ would [not] be complete without the mythical cutty, either ... stuck through a slit in the brim, or confined to the hat by a band” (*Duffy’s Hibernian Magazine*^1861^:282). Later in the 19th century, as physicians became increasingly aware of the health risks associated with tobacco consumption, it was argued that “if marriage were to be confined to the smokers, a physically inferior race of men and women would be begotten” (Oldberg and Helfman 1895), which gives further insight into how negative “racial” attributes came to be assigned to the Irish during this time.

Clay-pipe smoking was a common practice in the 19th century (J. Goodman 1994), but in Ireland the habit had different cultural connotations than on the neighboring island of Britain and elsewhere, which, in a way, explains how it eventually became a stereotypical Irish attribute. The smoking of clay pipes was an important element in the Irish funerary tradition of the 18th and 19th centuries (Mooney 1888; Ó Súilleabhaín 1969; Butler 2016). These pipes—or *dúidíní*, as they are called in Irish—would also sometimes be placed with the dead in the coffin, although no archaeological evidence of that practice was found at the Kilkenny Union Workhouse or at the Spike Island burial grounds. The absence of such evidence is not surprising at Spike Island, as convicts were not allowed access to tobacco. Smoking was also forbidden in the workhouse, according to the rules stated by the Irish Poor Law Commissioners (1849:appendix A, no. 3, article 20,31); however, the minute books from the Kilkenny Union reveal that this particular directive was not always followed. For instance, on 11 November 1848, a male inmate by the name of Edmund Delaney accidentally caused a fire to his coat and bed in the infirmary ward, and the Kilkenny Guardians demanded that the smoking ban be strictly enforced. This order was also a response to criticism from the Poor Law inspectors, who on numerous occasions had complained about the evidence of smoking inside the workhouse (Kilkenny Union Board of Guardians 1848).

**Fig. 9:**
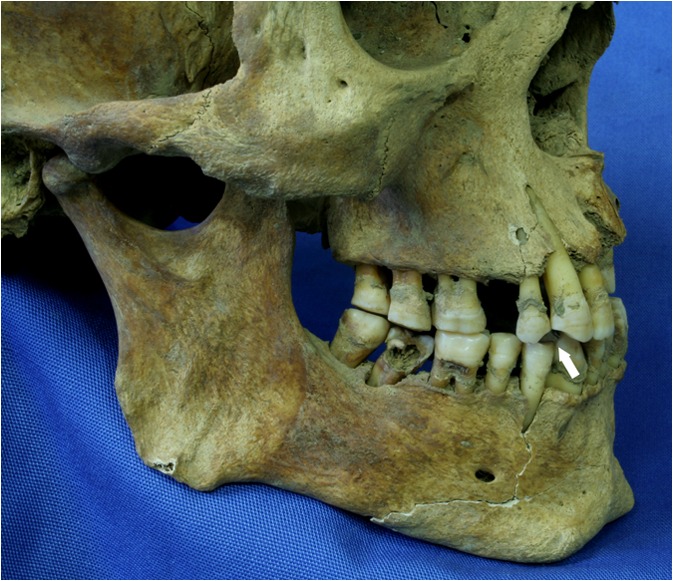
The dentition belonging to a middle-adult male from one of the Kilkenny Union Workhouse mass burials, exhibiting a clay-pipe facet (indicated by an arrow) as well as considerable evidence of dental disease. (Photo by Jonny Geber, 2009.)

That smoking was a common habit in both groups is evidenced by the very high prevalence in adult dentitions of pipe smokers’ notches, the typically circular abrasions on the anterior teeth that are due to wear caused by habitually clenching a pipe (Fig. 9). These were generally observed between the canines and first premolars on the left side of the mouth, which would have enabled smokers to free their right hands and, therefore, suggest that people were often smoking while engaged in other tasks. In Kilkenny, 61% (101/165) of all males and 29% (41/142) of all females had such facets (Geber 2015). Among the exclusively male convicts at Spike Island, the percentage with pipe-smoking notches was 77.3% (17/22). As smoking was forbidden in the prison and workhouse (although this was evidently not always adhered to, as noted above), this represents patterns of activity that predate institutionalization. These rates are substantially higher than in contemporary skeletal samples from England. For example, at Bow Baptist Church in London (1816–1837), only 2% of the males (2/86) had facets (Henderson et al. 2013), and at St. Marylebone (1767–1859), also in London, only 1% (1/105) of the males did (Miles et al. 2008). At a third London burial ground, St. Mary and St. Michael (1843–1854), nearly 40% (55/139) of all males, but only 3% (3/102) of females had facets (D. Walker and Henderson 2010). This particular cemetery, located at Whitechapel in the East End, was used by poorer Irish immigrants, and further implies that pipe smoking was an integral part of life for many people with an Irish identity during the 19th century.

A late 19th-century American commentator was of the opinion that upper middle-class and elite Irish men smoked less than their English counterparts (Billings 1875:209), but the opposite was clearly the case for the poor. Indeed, this popularity of tobacco use among the poor may explain why the wealthy Irish eschewed smoking, which may have been perceived as a badge of poverty. During a speech on taxation in the House of Commons on 26 March 1830, Lord Charles Edward Poulett Thompson, MP (1799–1841), stated that tobacco consumption in Ireland was significantly higher than in England, and that tobacco, in fact, was “a prime necessity amongst the lower orders in that country” (Poulett Scrope 1844:345). This “prime necessity” was perhaps related to the appetite-suppressive qualities of nicotine (Mineur et al. 2011). When seen in the context of colonial domination, however, the habit of pipe smoking in Ireland during the 19th century can also be viewed as a political act relating to social and national identity. In this view, smoking was a form of resistance and could be viewed in terms of a material expression of anticolonial discourse (Hartnett 2004). Not only did this take the shape of the habit of smoking, but, also, in the latter half of the 19th century, the pipe itself. These would often be adorned with nationalist imagery, such as shamrocks, harps, busts of political figures, or direct political mantras, such as “Erin go Bragh” (an Anglicization of the Irish *Éirinn go brách*, meaning “Ireland forever”), “Home Rule” (advocating self-governance for Ireland), or “Repeal” (referring to demands for the repeal of the Act of Union). Through this direct symbolism adorning the pipes themselves, a smoker could take an obvious political stance without having to resort to language that could be deemed seditious. These decorated pipes have been found outside Ireland, for instance in archaeological excavations of Irish tenement areas in the Five Points neighborhood in Lower Manhattan, New York City (Fox 2015:87–91). For those who left Ireland in the 19th century, the continuous use of the clay pipe in their country of adoption could have been a means of expressing their national identity (Cook 1997; Reckner and Brighton 1999).

## Discussion

In this article, we have taken the view that, despite the fact that Ireland was constitutionally integrated into the United Kingdom from 1801, other aspects of the British/Irish relationship, such as the almost annual Coercion Acts with the associated suspension of rights, as well as the presence of a social elite that was perceived to be distinct in terms of religion and ethnicity, indicate that the association between the two islands was one of colonial master and subject. In common with British colonialism in other world areas, the unequal relationship between the two islands and unequal social relations within Ireland were legitimized and sustained by narratives of racial distinctiveness and inferiority that were often articulated in terms of moral failure due to inherent flaws. These narratives of difference included myths about the stature of the Irish, their supposed innate violence, and their use of tobacco, parameters that are amenable to bioarchaeological investigation. These are also factors that were well-documented by contemporary observers, but the historical record in this regard is problematic, created as it was by those in positions of power and authority.

Bioarchaeology offers an alternative means of exploring the lives and deaths of the poorest cohorts of Victorian society, those who were silenced and marginalized. Through the conflation of race with poverty, the emphasis on the distinctiveness of the Irish and their inherent failings was a tool used in their political and economic subjugation. It also served to exonerate successive governments and their roles in the systematic pauperization of most of the population of the island. Bioarchaeological investigation of two aspects of this mythical distinctiveness, stature and interpersonal violence, showed that the differences, in respect of these metrics, between the Irish and their English peers were overstated. This was done for a strategic purpose, to facilitate and justify the domination of the island’s population. This colonial domination was resisted explicitly by various political and civic organizations throughout the 19th century. We suggest that the bioarchaeological evidence for tobacco consumption relates to an activity that was a means of expressing a particular identity and, in doing so, was a form of passive resistance, both intentionally and in a non-discursive manner. Smoking has often been a subversive activity.

Despite the rich historical legacy of the period—the Victorians were prodigious record keepers and publishers—relatively little is known of the 19th-century poor in terms of their actual experiences and the coping strategies they adopted for the dire conditions in which many of them lived. The bioarchaeology of the Kilkenny Union Workhouse and the Spike Island prison provides remarkable and emotive insights into what life was like in Ireland for those among the poor who found themselves institutionalized in the middle of the 19th century.

## Footnotes

1 The human remains from Kilkenny were reburied during a multidenominational ceremony at the Famine Memorial Garden in Kilkenny City in May 2010. The long-term fate of the Spike Island remains will be decided by the National Museum of Ireland, which has statutory responsibility for the curation of all archaeological artifacts (including archaeological human remains) in the state.

2 The osteological determination of living stature from skeletal remains is based on regression equations, denoted from a reference sample, which give an estimate within varying degrees of error margins. It is, therefore, not recommended to assess the results from these on an equal basis as anthropometrically measured data.
